# Influence of low dose of gamma radiation and storage on some vitamins and mineral elements of dried oyster mushrooms (*Pleurotus ostreatus*)

**DOI:** 10.1002/fsn3.432

**Published:** 2016-10-11

**Authors:** Nii Korley Kortei, George Tawia Odamtten, Mary Obodai, Michael Wiafe‐Kwagyan, Edward Adotey Addo

**Affiliations:** ^1^ Department of Nuclear Agriculture and Radiation ProcessingGraduate School of Nuclear and Allied SciencesUniversity of GhanaLegon, AccraGhana; ^2^ Department of Plant and Environmental BiologyCollege of Basic and Applied SciencesUniversity of GhanaLegonGhana; ^3^ Food Microbiology DivisionCouncil for Scientific and Industrial Research– Food Research InstituteAccraGhana; ^4^ Department of NutritionNoguchi Memorial Institute for Medical ResearchLegonGhana

**Keywords:** gamma irradiation, mineral elements, oyster mushrooms, *P. ostreatus*, storage, total soluble solids, vitamins

## Abstract

Mushrooms contain some of the most potent natural medicines on the planet. Vitamins A, C, D, Mineral elements, contents, as well as total soluble solids (Brix^o^) of dried composition of *Pleurotus ostreatus* were investigated after exposing to gamma radiation doses of 0 (control), 0.5, 1, 1.5, and 2 kGy at a dose rate of 1.7 kGy per hour in air from a Cobalt 60 source (SLL 515, Hungary) batch irradiator prior to storage (0 month) and after storage (12 months) at room temperature (28 ± 2°C). Results obtained showed some significant (*p* < .05) differences due to irradiation and storage. Before storage, vitamins A, C, and D contents of dried and irradiated mushrooms ranged 0.003 ± 0.08–0.014 ± 0.08, 0.042 ± 1.06–0.132 ± 1.06, and 0.040 ± 0.76–0.057 ± 0.76 mg/g, respectively. After 12 months, vitamin contents decreased and ranged 0.0029 ± 0.08–0.010 ± 0.08, 0.038 ± 1.06–0.125 ± 1.06, and 0.031 ± 0.76–0.05 ± 0.76 mg/g for vitamins A, C, and D, respectively. Total soluble solids recorded 1.5 Brix^o^, however, showed no significant difference (*p* > .05) and did not change in 12 months after gamma irradiation up to 2 kGy. Sodium ranged from 14.00 ± 0.7 to 14.90 ± 0.8 mg/100 g. Potassium content varied from 30.20 ± 0.5 to 33.10 ± 0.6 mg/100 g. Magnesium content ranged 1.27 ± 0.15–3.53 ± 0.04 mg/100 g. Calcium ranged 11.00 ± 0.4–12.53 ± 0.4 ± 0.03 mg/100 g. Phosphorus content ranged 6.11 ± 0.30–6.41 ± 0.35 mg/100 g, whereas Nitrogen content was found to be 3.00 ± 0.03–3.60 ± 0.25 mg/100 g. Microelements or heavy metals included Copper; detected ranged 0.00 ± 0.00–0.02 ± 0.001 mg/100 g, Zinc content ranged 0.01 ± 0.002–0.03 ± 0.001 mg/100 g. Iron content was found to be in the range 0.29 ± 0.01–0.37 ± 0.1 mg/100 g. Manganese content was found to be in the range 0.03 ± 0.001–0.04 ± 0.01 mg/100 g. Lead content was found to be 0.00 ± 0.00–0.03 ± 0.001 mg/100 g. Food processing and storage has the potential to slightly alter the stability of vitamins in foods. *Pleurotus ostreatus* showed appreciable levels of mineral elemental composition, essential vitamins A, C, and D, and can be endorsed as a natural medicinal food product in the food and pharmaceutical industries. The heavy metals detected were with also below the upper limits permissible by the WHO standards and is thus safe for human consumption.

## 1 Introduction

Oyster mushrooms (*Pleurotus* species) possess nutritive and medicinal attributes which dates back to ancient times as early as 1500 BC recorded in ancient literature. They are considered as functional foods because they elicit their positive effect on humans and animals (Buah, Van Der puije, Bediako, Abole, & Showemimo, 2010). Oyster mushrooms are the second largest commercially produced mushroom in the world (Van Nieuwenhuizen, 2009) and are cherished due to their culinary, nutritional, as well as medicinal properties (Barros, Ferreira, Queiros, Ferreira, & Baptista, 2007; Kalac, 2012; Singh, Patel, & Naraian, 2012). Nutritionally, they are considered as source of proteins, vitamins, fats, carbohydrates, amino acids, and minerals (Jiskani, 2001; Kortei & Wiafe‐ Kwagyan, 2015). Medically, *P. ostreatus* fruiting bodies and mycelia possess a myriad of therapeutic properties like anti‐inflammatory, immunomodulatory, anticancer activity, ribonuclease activity, antimicrobial, hypotensive, hyperglycemic, and antiviral with the potential to act against the Human Immunodeficiency Virus (HIV) (Li, Liu, Wang, & Ng, 2008; Wang & Ng, 2004).

Total Soluble Solids measure the sum of the solids which are in solution. As it increases, water activity is reduced and survival of microorganisms becomes less likely (The Science Dictionary, 2015. Data on mushroom polysaccharides have been collected from hundreds of different species of higher fungus; some specific carbohydrates with these properties have been quantified in different mushrooms: rhamnose, xylose, fucose, arabinose, fructose, glucose, mannose, mannitol, sucrose, maltose, and trehalose (Ferreira, Barros, & Abreu, 2009).

Food irradiation processes have been widely studied and are as well known as any other food processing method, such as dehydration and freezing (Arvanitoyannis, 2008; Crawford & Ruff, 1996). Nutritional value of the foods subjected to various processing techniques, especially food irradiation, has been questioned by both the activists and consumers alike (Crawford & Ruff, 1996; Kilcast, 1994). One of the main impedances for the development of this technique in many countries is the misconception consumers have with regards to excessive nutrient denaturation, along with the myth of food becoming radioactive and generation of toxic compounds (Kilcast, 1994). However, results of research as far back as the 1950's have already shown the absence of radioactivity inducement in the food treated by ionizing radiations (Wiendl, 1984).

Gamma irradiation as a means of preservation of foods has received endorsements by several international bodies (FAO, OIEA, OPS, 1992; ICGFI, 1991; International Atomic Energy Agency, 1999) as an effective means of decontamination which produces minimal or no loss in sensory attributes. The main advantages of irradiation are the small alterations in food components (Kilcast, 1994). Micronutrients, especially vitamins, can be susceptible to any food treatment method (Crawford & Ruff, 1996; WHO ‐ World Health Organization, 1994), but the extent of losses must not exceed the limit required in food for therapeutic and nutritional benefit to the consumer.

This study investigated the effect of gamma irradiation and storage time on the properties of some essential vitamins (A, C, D), mineral elements, and total soluble solids of *P. ostreatus*.

## 2 Materials and Methods

### 2.1 Mushroom material

*Pleurotus ostreatus* mushroom samples were grown on composted sawdust as described by Kortei et al. (2014) and harvested at maturity from the cropping house of the Mycology Unit, Food Research Institute, Accra, between the periods of February and May, 2014.

### 2.2 Processing

#### 2.2.1 Drying of mushroom samples and storage

Drying was carried out by using a solar dryer at a temperature of 50–60°C to reduce moisture content to about 12% for an average period of 12 days as prescribed by Kortei, et al. (2016), Kortei, Odamtten, Ayim‐ Akonor, and Akonor (2016), and Akonor and Tortoe (2014). The dried samples of the mushroom were stored in polythene and polypropylene packs before and after at room temperature.

#### 2.2.2 Irradiation of mushroom materials

Forty (40) grams of dried oyster mushrooms (*Pleurotus ostreatus)* were packed and irradiated at doses of 0 (control), 0.5, 1, 1.5, and 2 kGy at a dose rate of 1.7 kGy per hour in air at 28 ± 2°C from a Cobalt 60 source (SLL 515, Hungary) batch irradiator. Doses were confirmed using Fricke's dosimetry system which is a reference chemical dosimeter based on the chemical process of oxidation of ferrous ions (Fe^2+^) in aqueous sulfuric acid solution to ferric ions by ionizing radiation at the Radiation Technology Centre of the Ghana Atomic Energy Commission, Accra, Ghana.

### 2.3 Vitamin content analysis

#### 2.3.1 Provitamin A or beta carotene (standard preparation)

A small amount of the pure trans‐beta‐carotene was dissolved in petroleum ether and its concentration was spectrophotometrically determined using the formular below.

$$\begin{array}{cc} \text{Concentration (mg/w)} & =\frac{\text{Absorption}\times 10,000}{2592}\\ & =\frac{0.348\times 10,000}{2592}\\ & =1.3442\;\mu \text{g/ml} \end{array}$$

### 2.4 Samples preparation

Extraction was done with 50‐ml cold acetone in mortar using a pestle. It was repeated until the mushroom sample was devoid of color. The extracts were pooled and filtered. The filtrate was partitioned on a 20‐ml petroleum spirit in a 500‐ml separating funnel. It was washed several times with distilled water until the aqueous layer became clear. The petroleum spirit was dried by passing it through anhydrous sodium sulfate sitted on cotton wool at the base of the funnel. The total volume of the extract was recorded. The total volume, which is a representative of the sample weight, was evaporated under a stream of Nitrogen gas, reconstituted with 1 ml of the mobile phase and, finally, 20 μl was injected into the High Performance Liquid Chromatoghraph (HPLC) (Shimadzu SPD‐6A UV spectrophotometric detector, Japan).

The standard was injected thrice and the average of the three (3) corresponding areas was calculated.

The average standard Area = 141282
Therefore, 1.3442 μg/ml = 141282

The samples were injected and the respective areas were obtained using the formular below from which the respective concentrations were calculated:

$$\begin{array}{cc} \text{Microgram per gram}\;(\mu \text{g/g}) & =\\ & \frac{\text{Sample Area}\times \text{Standard Conc.}\;(\mu \text{g/ml})\times \text{Total Vol. Extract}}{\text{Std Area (Beta carotene)}\times \text{Sample Weight}} \end{array}$$

#### 2.4.1 Analysis of ascorbic acid

Ascorbic acid was analyzed by reversed‐phase chromatography with tetrabutylammonium added as an NH_2_ column.

##### Analytical conditions:

Column: NH12 P‐50 4 E (250 mml × 4.6 mm i.d.)
Mobile phase: A 100 mmol/L (Tremethanol amine) phosphate buffer (pH 2.2);B Acetonitrile
Flow rate: 1.0 ml/min
Column temp: 40°C
Detection: Ultra Violet DETECTOR
Wave length: 240 nm
Dilution: 50 times

##### Extraction

Extraction was done by ultrasonication and was diluted with the mobile phase. It was filtered through 0.45‐μm membrane filters and 20 μl was injected into the HPLC column.

##### Standard vitamin C

A known standard concentration (100 mg/Tab.Vitro C, Kinapharma, Ghana) was used to calibrate the instrument which in turn gave a factor, upon which all samples concentration was calculated.

#### 2.4.2 Analysis of vitamin D

This was carried out using the method prescribed by Wallace, Gibson, De La Hunty, Lamberg‐Allardt, and Ashwell (2010).

#### 2.4.3 Determination of total soluble solids

Estimation was done by dissolving 1 g of dried mushroom sample in 10‐ml distilled water and content of sample detected by a hand‐held optical refractometer (RF30, Extech Instruments, U.S.A).

#### 2.4.4 Determination of moisture content

The moisture content was determined by the gravimetric method of AOAC (1995).

#### 2.4.5 Determination of macro‐ and microelements (heavy metals)

This procedure was carried out according to a modified method of Obodai et al. (2014). Approximately 0.3 gram was weighed into labeled digestion tubes and dissolved in 2 ml concentrated HNO_3_. The solution was heated at 450°C for 4 hr and later dissolved in 1 ml concentrated H_2_SO_4_, 1 ml HNO_3_, and 1 ml H_2_O_2_, and then diluted with double deionized water up to a volume of 25 ml. A blank digest was carried out by following the above procedure. Contents of macroelements and microelements (heavy metals) in the mushroom samples were determined by using Atomic Absorption Spectrophotometer (Perkin Elmer precisely A Analyst 400).

## 3 Results and Discussion

According to Murano (1995), when food is irradiated, there is a reaction of ionizing radiation and water in the food causing the release of electrons and the formation of highly reactive free radicals. The free radicals interact with vitamins in ways that can alter and degrade their structure and/or activity. The results obtained from the analysis of total vitamins are presented in Tables 1 and 2. Initial vitamin A contents of mushroom stored ranged from 0.0038 ± 0.0007 to 0.012 ± 0.0008 mg/g. After 12 month of storage, it ranged 0.0031 ± 0.0009–0.010 ± 0.002 mg/g. Low gamma radiation doses and storage time had significant (*p* < .05) effect on vitamin A content of *P. ostreatus*. The extent to which vitamin loss occurs can vary based on a number of factors, including the type of food, temperature of irradiation, and availability of oxygen. Nonetheless, vitamin loss almost always increases with increasing doses of radiation (Kilcast, 1994).

**Table 1:** Effect of gamma irradiation on vitamin A, C, and D (mg/g) contents and total soluble solids (Brix^o^) of mushrooms before and immediately after irradiation

Dose (kGy)	Vitamin A (mg/g)	Vitamin C (mg/g)	Vitamin D (mg/g)	T.S.S Brix^o^
0	0.0068 ± 0.0003^b^	0.132 ± 0.001^c^	0.049 ± 0.004^a^	1.5 ± 0.0^cd^
0.5	0.01 ± 0.0035^c^	0.056 ± 0.005^b^	0.044 ± 0.003^a^	1.5 ± 0.0^cd^
1	0.005 ± 0.002^b^	0.088 ± 0.023^b^	0.050 ± 0.008^a^	1.5 ± 0.0^cd^
1.5	0.012 ± 0.0008^c^	0.046 ± 0.007^a^	0.054 ± 0.008^a^	1.5 ± 0.0^cd^
2	0.0038 ± 0.0007^a^	0.083 ± 0.008^c^	0.040 ± 0.009^a^	1.5 ± 0.0^cd^

Means (3) ± *SE* with different letter superscripts in a column are significantly different (*p* < .05).

**Table 2:** Effect of gamma irradiation on vitamin A, C, and D (mg/g) contents and total soluble solids (Brix^o^) of mushrooms stored for 12 months in polypropylene materials

Dose (kGy)	Vitamin A (mg/g)	Vitamin C (mg/g)	Vitamin D (mg/g)	T.S.S Brix^o^
0	0.0058 ± 0.0002^b^	0.125 ± 0.018^c^	0.041 ± 0.005^b^	1.5 ± 0.0^cd^
0.5	0.01 ± 0.004^c^	0.044 ± 0.003^b^	0.034 ± 0.0005^a^	1.5 ± 0.0^cd^
1	0.005 ± 0.002^b^	0.080 ± 0.02^b^	0.046 ± 0.008^b^	1.5 ± 0.0^cd^
1.5	0.010 ± 0.002^c^	0.042 ± 0.005^b^	0.042 ± 0.005^b^	1.5 ± 0.0^cd^
2	0.0031 ± 0.0009^a^	0.075 ± 0.008^b^	0.036 ± 0.007^a^	1.5 ± 0.0^cd^

Means (3) ± *SE* with different letter superscripts in a column are significantly different (*p* < .05).

Vitamin A is necessary for clear vision in dim light. It also maintains the integrity of epithelial tissue (Gopalan et al., 2000). The range of results obtained in this study was similar to results reported by Musieba, Okoth, Mibey, Wanjiku, and Morsa (2013) who investigated the proximate composition, amino acids, and vitamins profile of *Pleurotus citrinopileatus* in Kenya. Kumari and Achal (2008) reported vitamin A contents of 0.282 ± 0.004 mg/g for dry fruit body; 0.363 ± 0.004 mg/g fresh fruit body mg/g. Results obtained for vitamin A in this study contrast the results of Jonathan, Okon, Oyelakin, and Oluranti (2012) who reported absence (0.00 mg/100 g) of vitamin A in dry *P. ostreatus* mushrooms cultivated on various substrates of cotton wastes, rice straw, and sawdust.

Vitamin C (ascorbic acid) contents of mushrooms stored initially ranged from 0.046 ± 0.007 to 0.132 ± 0.001 mg/g. After 12 months storage, it ranged from 0.042 ± 0.005 to 0.125 ± 0.018 mg/g. Generally, gamma radiation and storage time showed an apparent significant (*p* < .05) effect on ascorbic acid content. Mushrooms have been reported to have antioxidant activity which is correlated with their phenolic and polysaccharide compounds (Dubost, Ou, & Beelman, 2007)**.** The global economic value of mushrooms and their consumption is a combination of their value as food and their nutraceutical properties (Ferreira, Vaz, Vasconcelos, & Martins., 2010; Kortei & Wiafe‐ Kwagyan, 2015). The major antioxidants found in mushrooms are phenolic compounds, whereas other potential antioxidants, for example, vitamin C, β‐ carotene, and γ‐ tocopherols, have been found in small quantities (Yang, Lin, & Mau, 2002). Generally, irradiation dose of 0.5 kGy increased production of phenolics in *P. ostreatus* and caused a significant (*p* < .05) higher contents of phenolics (Kortei, et al. 2016, Kortei, Odamtten, Ayim‐ Akonor, et al. (2016)). On the other hand, 2 kGy recorded the least phenols in ethanol, methanol, and aqueous extracts of *P. ostreatus*. This may partly explain the decline in vitamin C with increase in dose from 0 to 2 kGy (Tables 1 and 2).

Vitamin C acts as the first‐line natural antioxidant and also serves as a free radical scavenger (Maxwell, 1995). The nonirradiated mushrooms recorded higher values. Previous studies by Kumari and Achal (2008) reported vitamin C values of 0.277 ± 0.0015 mg/g dry fruit body and 0.363 ± 0.0025 mg/g fresh fruit body when they studied the effect of different substrates on the production and nonenzymatic antioxidant activity of *P. ostreatus*. Obodai (1992) found the ascorbic acid (vitamin C) contents of some *Pleurotus* species as follow: *P. sajor‐caju* (Hong Kong) 11340 mg/100 g, *P. sajor‐caju* (Mauritius) 92.66 mg/100 g, *P. ostreatus* (EM‐ 1) 99.83 mg/100 g, and *Volvariella volvacea* (62.14 mg/100 g) all grown on sawdust (*Triplochiton scleroxylon*). Muthangya, Mshandete, Amana, Hashim, and Kivaisi (2014), however, reported values within the range from 5.07 ± 0.04 mg/100 g to 5.29 ± 0.02 mg/100 g in the *Pleurotus* HK 37 grown on *Agave sisalana* saline solid wastes. Jonathan et al. (2012) found vitamin C values of range 3.27 ± 0.47–3.65 ± 0.17 mg/100 g in *P. ostreatus* on different substrates. It is well known that vitamin C is the most sensitive of all water soluble vitamins to an irradiation (Kilcast, 1994). However, it has been noted that when reporting vitamin C levels in irradiated food, many workers have not taken into consideration the fact that ionizing radiation can cause a partial conversion of ascorbic acid into dehydroascorbic acid (Kilcast, 1994) reflecting in a lower content of ascorbic acid after irradiation.

Vitamin D content of mushrooms initially ranged from 0.040 ± 0.0090 to 0.054 ± 0.008 mg/g. Gamma radiation and storage time had no significant (*p* > .05) effect. After 12‐month storage, values ranging from 0.036 ± 0.007 to 0.046 ± 0.008 mg/g were detected. Jonathan et al. (2012) reported vitamin D values ranging 3.80 ± 0.12–4.22 ± 0.53 mg/100 g in *P. ostreatus* cultivated on various substrates such as cotton wastes, rice straw, and sawdust. Another study, Mattila et al. (2001) reported values of 0.3 μg/100 g in *P. ostreatus*. Vitamin D is particularly confusing in mushrooms and other fungal foods.

The initial total soluble solids recorded 1.5 ± 0.0 Brix^o^ did not change after 12 months of storage. There was no significant (*p* > .05) difference recorded during and after storage. Total soluble solid was earlier reported to be the major respiration substrate in *A. bisporus* during postharvest storage (Hammond & Nichols, 1975), and steady decreases in the soluble solids concentration were previously reported in fruit bodies stored at cold temperatures (Tseng & Mau, 1999). Nonetheless, radiation effects on TSS in mushrooms have not been reported exhaustively. This is the first report of effect of gamma irradiation on the TSS content of *P. ostreatus* cultivated in sawdust in Ghana. Total soluble solids did not change with increasing dose up to 2 kGy and storage for up to 12 months.

Mushrooms have a very effective bioaccumulation mechanisms which make them take up mineral elements from the ecosystem (Zhu et al., 2011). In this study, calcium content was found to be 11.00 ± 0.4–12.53 ± 0.4 ± 0.03 mg/100 g. There was statistical differences (*p* < .05) observed with respect to the different doses applied (Table 3 and 4). Applied dose of 2 kGy showed significance (*p* < .05) presumably due to its ability to stimulate the tissues of dried fruit bodies and activate enzymatic activities according to nutrient composition. Results obtained agree with reported values of 13.03 mg/100 g by Okechukwu, Okereke, Onyedineke, and Obi (2011). Oyetayo and Ariyo (2013) recorded values of 5.37 ± 0.01–8.87 ± 0.006 mg/100 g; Alam et al. (2008) detected values of 35.9 ± 3.8 mg/100 g for calcium in *P. ostreatus, P. sajor‐caju*,* P. florida*, and *Calocybe indica*. Calcium aids in formation of strong bones and teeth (USDA, 2010) and is found in adequate quantities in the fruit body of *P. ostreatus*.

**Table 3:** Effect of irradiation on the elemental composition of *P. ostreatus* before storage (0 months)

Element (mg/100 g)	Dose Applied (kGy)				
	0	0.5	1.0	1.5	2.0
Calcium	11.00 ± 0.3^a^	11.03 ± 0.4^a^	11.85 ± 0.5^b^	11.32 ± 0.3^b^	12.20 ± 0.4^c^
Potassium	31.91 ± 0.5^b^	30.20 ± 0.5^a^	30.72 ± 0.5^a^	32.84 ± 0.6^c^	33.10 ± 0.6^c^
Magnesium	1.77 ± 0.18^a^	1.27 ± 0.15^a^	2.40 ± 0.05^b^	2.40 ± 0.05^b^	3.53 ± 0.04^c^
Nitrogen	3.51 ± 0.02^c^	3.56 ± 0.02^c^	3.31 ± 0.04^b^	3.00 ± 0.03^a^	3.59 ± 0.05^a^
Phosphorus	6.10 ± 0.35^bc^	6.14 ± 0.35^b^	6.11 ± 0.30^a^	6.27 ± 0.25^b^	6.32 ± 0.25^b^
Sodium	14.10 ± 0.7^a^	14.10 ± 0.8^a^	14.61 ± 0.8^b^	14.00 ± 0.7^a^	14.90 ± 0.8^bc^
Zinc	0.03 ± 0.001^b^	0.03 ± 0.001^b^	0.01 ± 0.002^a^	0.01 ± 0.002^a^	0.01 ± 0.002^a^
Manganese	0.04 ± 0.001^b^	0.03 ± 0.001^a^	0.03 ± 0.001^a^	0.04 ± 0.001^b^	0.04 ± 0.002^b^
Lead	0.02 ± 0.001^b^	0.02 ± 0.001^b^	0.02 ± 0.001^b^	0.03 ± 0.001^bc^	0.00 ± 0.00^a^
Iron	0.37 ± 0.1^c^	0.31 ± 0.01^b^	0.35 ± 0.0^c^	0.29 ± 0.01^b^	0.35 ± 0.03^c^
Copper	0.02 ± 0.001^b^	0.02 ± 0.001^b^	0.01 ± 0.00^a^	0.02 ± 0.001^b^	0.00 ± 0.00^a^

Means ± *SE* with same letters in a row are not significantly (*p* > .05) different.

**Table 4:** Effect of irradiation on the elemental composition of *P. ostreatus* during storage up to 12 months

Element (mg/100 g)	Dose Applied (kGy)				
	0	0.5	1.0	1.5	2.0
Calcium	11.02 ± 0.3^a^	11.00 ± 0.4^a^	11.75 ± 0.5^b^	11.34 ± 0.3^b^	12.53 ± 0.4^c^
Potassium	31.91 ± 0.5^b^	30.20 ± 0.5^a^	30.72 ± 0.5^a^	32.84 ± 0.6^c^	33.10 ± 0.6^c^
Magnesium	1.77 ± 0.18^a^	1.27 ± 0.15^a^	2.40 ± 0.05^b^	2.40 ± 0.05^b^	3.53 ± 0.04^c^
Nitrogen	3.61 ± 0.02^c^	3.51 ± 0.02^c^	3.31 ± 0.04^b^	3.00 ± 0.03^a^	3.59 ± 0.05^a^
Phosphorus	6.41 ± 0.35^bc^	6.34 ± 0.35^b^	6.11 ± 0.30^a^	6.27 ± 0.25^b^	6.32 ± 0.25^b^
Sodium	14.10 ± 0.7^a^	14.50 ± 0.8^b^	14.67 ± 0.8^b^	14.00 ± 0.7^a^	14.90 ± 0.8^bc^
Zinc	0.03 ± 0.001^b^	0.03 ± 0.001^b^	0.01 ± 0.002^a^	0.01 ± 0.002^a^	0.01 ± 0.002^a^
Manganese	0.04 ± 0.001^b^	0.03 ± 0.001^a^	0.03 ± 0.001^a^	0.04 ± 0.001^b^	0.04 ± 0.002^b^
Lead	0.02 ± 0.001^b^	0.02 ± 0.001^b^	0.02 ± 0.001^b^	0.03 ± 0.001^bc^	0.00 ± 0.00^a^
Iron	0.37 ± 0.1^c^	0.31 ± 0.01^b^	0.35 ± 0.0^c^	0.29 ± 0.01^b^	0.35 ± 0.03^c^
Copper	02 ± 0.001^b^	0.02 ± 0.001^b^	0.01 ± 0.00^a^	0.02 ± 0.001^b^	0.00 ± 0.00^a^

Means ± *SE* with same letters in a row are not significantly (*p* > .05) different.

Potassium concentration levels were high in this study in the fruit bodies of *P. ostreatus* and ranged from 30.20 ± 0.5 to 33.10 ± 0.6 mg/100 g. There were significant differences (*p* < .05) with increasing radiation (up to 2 kGy) treatment (Tables 3 and 4). Obodai et al. (2014) reported values of range 7.40 ± 0.01–7.80 ± 0.05 mg/kg. Oyetayo and Ariyo (2013) reported values of the range 9.42 ± 0.15–11.34 ± 0.02 mg/100 g. Musieba et al. (2013) reported values of 2.28 ± 0.14 mg/100 g. *Pleurotus spp*. contains 182–395 mg/100 g (0.0182–0.0395 mg/kg) which is 3–11% of the Daily Value (USDA, 2010). Recommended Daily Intake (RDI) of potassium is 3100 mg/day (Manzi, Gambelli, Mariconi, Vivanti, & Pizzoferrato, 1999). Potassium aids in the maintenance of normal fluid and mineral balance in the control of blood pressure. It also plays a role in making sure nerves and muscles, including the heart, function properly (Duyff, 2006).

Magnesium content was found to be in the range 1.27 ± 0.15–3.53 ± 0.04 mg/100 g. There were significant differences (*p* < .05) observed with increasing dosages (Table 4). Levels obtained in this study fell within range of previous studies which were 1.69 ± 0.015–3.57 ± 0.01 mg/kg (Oyetayo & Ariyo, 2013), 1.067–1.380 mg/kg (Wiafe‐ Kwagyan, 2014), and 0.07 mg/100 g (Musieba et al., 2013).

Nitrogen content was found to be 3.00 ± 0.03–3.60 ± 0.25 mg/100 g. There were statistical differences (*p* > .05) observed with the varying treatment. The body utilizes nitrogen for promoting protein synthesis, the creation of compounds and amino acids influence growth, hormones, brain functions, and the immune system. About 0.83 gram of protein per kilogram per day is considered sufficient to cover nitrogen requirements (World Health Organization, 1982a,b). Ahmed, Abdullah, Ahmed, and Borhannuddin Bhuyan (2013) reported a range of nitrogen 45–49 mg/kg in oyster mushrooms in Bangladesh. Recently, Layman (2013) suggested a maximum intake of 2–2.5 g/kg of body weight per day.

Phosphorus content was found to be 6.11 ± 0.30–6.41 ± 0.35 mg/100 g. There were significant differences (*p* < .05) observed with increasing radiation. Dose of 2 kGy had an apparent increasing effect on phosphorus in the dry tissues of mushroom fruit bodies. Presumably, the higher doses stimulated enzyme activities. Phosphorus concentration obtained fell within range of values recorded in *Pleurotus sp*. by Ahmed et al. (2013) who reported values 8–9 mg/kg; Wiafe‐ Kwagyan, Obodai, Odamtten, and Kortei (2016) also recorded a range of 6.31–10.07 mg/kg in *P. eous*. Baig, Syed, Kadam, Mane, and Patil (2010) also recorded a range of 7.90–9.10 mg/100 g. As RDI of P is 0.7 g, *P. ostreatus* is high in P content, and can therefore contribute to human nutrition as good source of phosphorus (Çağlarirmak, 2007).

Sodium contents in this study ranged from 14.00 ± 0.7 to 14.90 ± 0.8 mg/100 g. There were significant differences (*p* < .05) with increasing dosage. The preponderance of mineral elements in the fruit bodies could be attributed to the varying degrees of stimulatory effect of doses on dry matter due to activation of cellular and extracellular metabolic enzymes (Dawoud & Abu Taleb, 2011). Oyetayo and Ariyo (2013) reported values of range 4.03 ± 0.02–4.39 ± 0.012 mg/kg in *P. ostreatus*. Regula and Siwulski (2007) recorded values of range 3.7 ± 21.4 mg/kg and Obodai et al. (2014) recorded values of 3.80 ± 0.01 mg/kg. According to Mallikarjuna et al. (2013), sodium is good for patients with hypertension, however, relatively less amounts are needed. Data from this work make *Pleurotus ostreatus* a good source of sodium for treatment of hypertension and is recommended for consumption.

Some heavy metals such as Zinc (Zn), Manganese (Mn), Lead (Pb), Iron (Fe), and Copper (Cu) were detected in the dry unirradiated and irradiated fruit bodies of *P. ostreatus* albeit in very minute quantities. Heavy metal concentration in mushrooms is considered higher than those in agricultural crop plants, vegetables, and fruits. This connotes that mushrooms have a very effective mechanism which enables them to readily take up some heavy metals from the environment (Zhu et al., 2011) due to their dense mycelia system which infiltrates the substrate (García, Alonso, & Melgar, 2005).

Zinc content was found to be in the range 0.01 ± 0.002–0.03 ± 0.001 mg/100 g (Tables 3 and 4). There was significant differences (*p* < .05) observed with doses applied. Zinc levels obtained in this study were within the RDI of trace elements reported by Indian Council of Medical Research (ICMR) (1990). Soylak, Saracoglu, Tȕzen, and Mendli (2005) recorded values of range 45.2–173.8 mg/kg, Tuzen (2003) recorded a range 33.5–89.5 mg/kg, and Isiloglu, Yilmaz, and Merdivan (2001) also recorded a range of 29.3–158 mg/kg. Zn is an essential micronutrient associated with a number of enzymes, especially in the synthesis of ribonucleic acids and DNA polymerases (Sadiq, Bhatti, & Hanif, 2008).

Manganese content was found to be in the range 0.03 ± 0.001–0.04 ± 0.01 mg/100 g. Manganese plays an important role in enzymatic catalysis and is crucial to virtually all biochemical and physiological process (Sadiq et al., 2008). Ahmed et al. (2013) obtained a range of 2.3 ± 0.1–2.6 ± 0.1 mg/kg, Soylak et al. (2005) obtained results ranging 14.2–69.7 mg/kg, while Tuzen (2003) obtained results ranging from 12.9–93.3 mg/kg. Sesli and Tüzen (1999) also obtained results ranging from 14.5 to 63.6 mg/kg in macrofungi in Turkey. Results obtained in this study were within the RDI of trace elements reported by Indian Council of Medical Research (ICMR) (1990) and was also found to be below toxicity levels of 400–1000 mg/kg (World Health Organization, 1982a,b).

Lead concentrations of *P. ostreatus* were found to be nil (0.00 ± 0.00–0.03 ± 0.001 mg/100 g). Results obtained agreed with levels reported by Regula and Siwulski (2007) who did not find lead in *Pleurotus ostreatus* and *Lentinus edodes*. Tuzen (2003) and Tuzen, Özdemir, and Demirbas (1998) obtained values of range 0.75–7.77 mg/kg. Wiafe‐ Kwagyan (2014) recently recorded 0.004 mg/kg and nil to 0.202 mg/kg in *P. eous* (Wiafe‐ Kwagyan et al., 2016). According to FAO/WHO (2001) tolerable weekly intake of lead is 0.025 mg/kg body weight. Lead (Pb) is toxic even at trace levels (Dobaradaren, Kaddafi, Nazmara, & Ghaedi, 2010) and the impairment related to Pb toxicity in humans includes abnormal size and hemoglobin content of the erythrocytes, hyperstimulation of erythropoisis and inhibition of hemoglobin synthesis. Lead concentrations detected in *P. ostreatus* in this study were very low and is considered safe for human consumption.

Iron content in this study was found to be in the range 0.29 ± 0.01–0.37 ± 0.1 mg/100 g. Values obtained in this study were lower than results reported by Regula and Siwulski (2007) who recorded 68.6 ± 5.50 mg/kg. Tuzen (2003) recorded 146–835 mg/kg, Sesli and Tüzen (1999) found 31.3–1190 mg/kg, and Isiloglu et al. (2001) also recorded 180–407 mg/kg. Main functions of iron include transport and storage of oxygen which aids in energy production and cell diffusion. It helps the immune and central nervous systems. Iron is the only nutrient for which women have a higher daily requirement than men. The U.S. Recommended Daily Allowance (RDA) of iron for men is 10 milligrams and 15 milligrams for women. According to Mamashealth (2013), breastfeeding increases iron requirement by about 0.5 to 1.0 mg a day.

Copper content was found to be nil (0.00 ± 0.00–0.02 ± 0.001 mg/100 g) (Tables 3 and 4). There were statistical differences (*p* < .05) observed with increasing radiation doses. Levels of copper obtained in this study were below the safe limit set by World Health Organization (WHO) (40 mg/kg) as copper in foods (World Health Organization, 1982a,b). Copper levels in mushrooms reported by some researchers were 4.71–51.0 mg/kg (Tuzen et al., 1998); 13.4–50.6 mg/kg (Soylak et al., 2005); 12–181 mg/kg (Tuzen, 2003); and 0.0018–0.08 mg/kg (Wiafe‐ Kwagyan, 2014). Copper is an essential constituent of some metallo‐enzymes and is required in hemoglobin synthesis in red blood cells which carry oxygen throughout the body. It helps keep bones and nerves healthy (Duyff, 2006) and aids in the catalysis of metabolic growth (Silvestre, Lagarda, Farra, Martineze‐Costa, & Brines, 2000).

## 4 Conclusion

The primary effects of radiation on vitamins at low and medium doses are not considerable. Studies showed that after low‐dose gamma‐irradiation, vitamin losses in the food were minimal in most cases. Mineral elements found in this study were below the WHO prescribed safe limits and so are safe for human consumption. Although the fruit bodies contained heavy metals like Zn, Fe, Mn, Pb, and Cu, their concentrations were below safe limits set by the WHO and render *P. ostreatus* safe for human consumption.

A regular and judicious consumption of this mushroom will be beneficial as nutrients and natural medicine giving a healthy diet to Ghanaians and most West African countries where these mushrooms are consumed on a regular basis.

## Conflict of Interest

None declared.
